# Sensing by Molecularly Imprinted Polymer: Evaluation of the Binding Properties with Different Techniques

**DOI:** 10.3390/s19061344

**Published:** 2019-03-18

**Authors:** Maria Pesavento, Simone Marchetti, Letizia De Maria, Luigi Zeni, Nunzio Cennamo

**Affiliations:** 1 Department of Chemistry, University of Pavia, 27100 Pavia PV, Italy; simone.marchetti01@universitadipavia.it; 2 Research on the Energetic System, 20134 Milan, Italy; Letizia.DeMaria@rse-web.it; 3 Department of Engineering, University of Campania Luigi Vanvitelli, 81031 Aversa, Italy; luigi.zeni@unicampania.it (L.Z.); nunzio.cennamo@unicampania.it (N.C.)

**Keywords:** molecularly imprinted polymer, synthetic biomimetic receptors, 2-furaldehyde, binding isotherms, electrochemical sensing tools, surface plasmon resonance sensing tools

## Abstract

The possibility of investigating the binding properties of the same molecularly imprinted polymer (MIP), most probably heterogeneous, at various concentration levels by different methods such as batch equilibration and sensing, is examined, considering two kinds of sensors, based respectively on electrochemical and surface plasmon resonance (SPR) transduction. As a proof of principle, the considered MIP was obtained by non-covalent molecular imprinting of 2-furaldehyde (2-FAL). It has been found that different concentration ranges of 2-FAL in aqueous matrices can be measured by the two sensing methods. The SPR sensor responds in a concentration range from 1 × 10^−4^ M down to about 1 × 10^−7^ M, while the electrochemical sensor from about 5 × 10^−6^ M up to about 9 × 10^−3^ M. The binding isotherms have been fit to the Langmuir adsorption model, in order to evaluate the association constant. Three kinds of sites with different affinity for 2-FAL have been detected. The sites at low affinity are similar to the interaction sites of the corresponding NIP since they have a similar association constant. This is near to the affinity evaluated by batch equilibration too. The same association constant has been evaluated in the same concentration range. The sensing methods have been demonstrated to be very convenient for the characterization of the binding properties of MIP in comparison to the batch equilibration, in terms of reproducibility and low amount of material required for the investigation.

## 1. Introduction

Chemical sensors are based on the strict integration of a receptor with an instrument able to generate a signal upon the combination of a substrate with the receptor. The characteristics of the binding reaction, in particular the affinity of the receptor for the substrate, are of overwhelming relevance for determining the performance of a sensor. Conversely, sensors can be used to investigate the combination of the receptor with the substrate. For example surface plasmon resonance (SPR) has been proposed as transduction method for a number of sensing applications since more than two decades ago [1], however it has been even more widely applied to evaluate the equilibrium and kinetical characteristics of the ligand-substrate interaction in investigations of biochemical and pharmaceutical interest [2,3,4]. Actually SPR is considered as one of the most robust and sensitive biosensing techniques that have been implemented in pharmaceutical research worldwide. The reason is that the detection based on SPR transduction is typically marker-free, thus it makes it possible the determination of the kinetics of molecular interactions, and of the equilibrium constants [5,6] for a wide variety of systems in real time, without the need for a molecular tag or label [7,8]. The SPR transduction is widely used for investigating the binding properties of bioreceptors as for example antibodies or aptamers, and for synthetic receptors like molecularly imprinted polymers (MIPs) [4,9]. Most often expensive dedicated apparatuses are used for binding investigation, based on the Kretschmann configuration [2,4].

In the present study we examine the possibility of using SPR transduction for the characterization of the binding properties of a receptor layer using a non-conventional SPR sensor based on optical fiber [10]. The SPR platform used in the present investigation has been previously proposed by our research group [11,12,13] and is based on a plastic optical fiber (POF) with a characteristic D-shape, i.e., with a planar surface obtained by erasing the cladding and partially the core of the optical fiber, over which a multilayer structure was realized as previously described. It is suitable for investigating the binding properties of the MIP since the signal obtained, i.e., the variation of the resonance wavelength (Δλ) is due to the variation of the refractive index of the polymeric layer, which depends on the binding of the substrate. Thus the device can be applied to investigate the receptor layer adsorbing properties, as it is often done by SPR measurement based on prism (Kretschmann configuration). Incorporating optical fiber, the cost of the sensor and the dimension of the apparatus are considerably reduced with respect to prism based configuration. Moreover the optical platform considered is easy to prepare directly on the POF core [12,13].

As a proof of principle, in the present study we investigated a synthetic receptor for a small molecule, 2-furaldehyde (2-FAL), obtained by molecular imprinting (MIP-2FAL). Receptors obtained by the molecular imprinting method, have been widely demonstrated to be advantageous with respect to biological receptors, in particular in the field of sensing, in terms of reproducibility, fast and low cost development, stability in time and possibility of application in chemically and biologically aggressive environment [14,15,16,17]. For this reason the investigation of the binding properties of these materials is of wide interest for sensing applications. The evaluation of the binding properties of MIPs can be a challenging task, due to the existence of heterogeneous binding sites in MIPs, particularly those synthesized by a non-covalent bulk procedure [18,19,20,21]. The heterogeneity may diminish the performance of MIPs in some analytical application. For example, it can contribute to broad, asymmetric peaks in chromatographic applications [22,23] and cross-reactivity in binding assays [23]. In any case it is important to recognize the heterogeneity and to characterize the different sites. This is commonly done by determining the binding isotherms in given concentration ranges depending on the detection or transduction technique considered. Then a model is fit to the binding isotherm to enable the estimation of the binding parameters. The most commonly used ones are the Langmuir and Langmuir-Freundlich isotherms [24,25,26,27,28,29,30,31]. The binding isotherms are most often obtained by equilibrating the solid with a solution containing the substrate and determining the concentration of the target substance in solution after equilibration with various methods [32]. This approach is quite demanding in terms of amount of solid required and complexity of the experimental procedure. In the present investigation the binding isotherms of 2-FAL on a MIP prepared by non-covalent imprinting have been determined not only by batch equilibration but also by the SPR sensor based on POF. The sensing method could improve the detection limits with respect to the batch equilibration, due to the fact that the template is preconcentrated at the electrode surface when it combines with the imprinted sites.

For the sake of comparison, the binding isotherms of 2-FAL on the corresponding MIP have been obtained also with a sensor based on a completely different transduction principle, i.e., electrochemistry.

Electrochemical sensors are chemically modified electrodes at the surface of which the species capable of specifically interacting with the desired analyte are fixed [33,34,35,36]. Various MIP based electrochemical sensors have been developed in the last years [35]. Our research in this field was focused on devices created by depositing a thin MIP layer over a screen printed electrochemical cell (SPC), so obtaining sensors for the determination of small molecules by different electrochemical techniques [36,37,38]. The MIP layer used in all these devices is easily and rapidly prepared, so it is particularly suitable to be used for rapid characterization purposes. The sensor developed in this investigation gives a voltammetric signal, generated by the substrate itself, i.e., 2-FAL, which is electroactive [39].

Sensing by different transduction methods would make it possible to examine the binding of 2-FAL to the same MIP in different concentration ranges, and to compare the results obtained. 2-FAL has been selected as a target molecule, as a proof of principle, for the development of an SPR-POF sensor for a small molecule, a kind of device which is sometimes believed to not be sensitive enough for practical application [17]. On the other hand, the development of a sensor for furanic compounds has an interest for controls in several fields, as in food technology and in biomass for energy production.

## 2. Materials and Methods

### 2.1. Reagents and Materials

Methacrylic acid (MAA) [79–41–4] (cat. no. M0782, Sigma-Aldrich, Saint Louis, MO, USA) and divinyl benzene (DVB) [97–90–5] (cat. no. 414565, Sigma-Aldrich) were filtered through an extraction column composed of 500 mg of aluminium oxide (Sigma-Aldrich, Saint Louis, MO, USA) for separation prior to use, in order to remove stabilizers. 2,2′-Azobisisobutyronitrile [78–67–1] (AIBN) was used as obtained from Sigma-Aldrich. All other chemicals were of analytical reagent grade. Solutions were prepared with ultrapure water (Milli-Q, Merck KGaA, Darmstadt, Germany). Screen-printed cells (SPC) with graphite working electrode (WE), graphite auxiliary electrode (AE), and Ag ink quasi-reference electrode (RE) on polyester support were commercially available (EcoBioServices s.r.l., Florence, Italy). Each cell was cut out from a strip of ten screen-printed cells to a width of 0.9 cm.

### 2.2. Prepolymeric Mixture

MIP was prepared by a non-covalent method as previously reported in many cases, for example in the case of trinitrotoluene and dopamine imprinted polymers [36,37]. A polymer with a similar composition has been prepared for 2-FAL too [12]. Briefly, the reagent mixture was composed of 30 μL of MAA, 665 μL of DVB, 10 μL of 2-FAL, and 15 mg of AIBN with molar ratio 1:4:40 2-FAL/MAA/DVB, without any solvent. The mixture was uniformly dispersed by sonication (visually homogeneous solution) and de-aerated with nitrogen for 10 min before polymerization. An identical prepolymeric mixture but not containing the template was prepared to synthesize a non-imprinted polymer (NIP) analogous to MIP.

### 2.3. Preparation of MIP Particles

The prepolymeric mixture was polymerized in an open tube in a thermostatic oven at 75 °C for 12 h. A solid with a glassy appearance was obtained. It was ground in a mortar to obtain particles of 100–300 μm diameter, which were dimensionally separated by sieving and washed for three times with fresh portions of 96% ethanol, at solvent/MIP ratio equal to 75 mL_sol_/ g_MIP_. The effectiveness of the extraction was verified by UV determination in the supernatant solution. A high amount of substances adsorbing at wavelength lower than 250 nm is extracted, however they do not interfere with the UV determination of 2-FAL, since their absorbance at 278 nm, which is the absorption maximum of 2-FAL, is very low. A final washing with water was then made in order to extract more polar fragments.

### 2.4. Preparation of the Polymer-Modified Electrochemical Cell

The commercially obtained SPC was washed in 96% ethanol and dried in an oven at 75 °C. Two sensors have been prepared as previously described [36], with different amounts of prepolymeric mixture. In one case (sensor SPC-MIP6) a volume of 8 μL of liquid polymeric mixture was dropped over the distal part of SPC, and in the other only 3 μL (sensor SPC-MIP5). In both cases an area of 0.3(±0.1) cm^2^, including all three electrodes, was covered by the liquid. This was polymerized in the air in a thermostatic oven at 75 °C for 12 h. After the polymerization, the cell was contacted for 1 h for three times with 5 mL of 96% ethanol to extract 2-FAL and unreacted monomers and stored in ethanol. NIP-modified cells were prepared by the same procedure, using the NIP prepolymeric mixture. The MIP-modified cell was stored in 96% ethanol. A NIP modified cell (SPC-NIP) analogous to SPC-MIP6 was prepared in the same way, but using NIP instead of MIP.

### 2.5. Preparation of the Polymer-Modified SPR Platform

The SPR optical platform was prepared as previously described [11,12,13]. Briefly, the plastic optical fiber (PMMA, with a 980 µm core and 20 µm thick fluorinated polymer cladding), without protective jacket, was embedded in a resin block 1 cm long, and the cladding was removed along half circumference together with part of the core, giving the characteristic D–shape to the device. The sensing region was about 10 mm long. Successively a layer of Microposit S1813 photoresist was spin coated on the planar region to obtain a 1.5 μm thick layer [11]. The refractive index, in the visible range of interest, is 1.49 for PMMA, 1.41 for fluorinated polymer and 1.61 for Microposit S1813 photoresist. Finally, a thin gold film (60 nm) was sputtered on platforms by a sputtering machine (Bal-Tec SCD 500, Leica, Wetzlar, Germany).

The gold planar surface over POF (SPR active surface) was washed with ethanol and then dried in a thermostatic oven at 60 °C prior to deposition of the sensing layer, i.e., the specific molecularly imprinted polymer (MIP) layer. About 40 μL of prepolymeric mixture were dropped over the platform, and spun for 60 s at 750 rpm. The polymerization was carried out exactly as described in the case of the sensor for electrochemical investigation, in the air in a thermostatic oven at 75 °C for 14 h. After the polymerization, the MIP layer was repeatedly washed with drops of 96% ethanol and flushing, to extract 2-FAL and unreacted monomers, and stored in air. This platform is indicated as D-shaped POF-MIP-SPR.

### 2.6. Experimental Methods for Binding Investigation

#### 2.6.1. UV Determination for Batch Equilibration

UV spectra of the considered furanic compounds are shown in the Supplementary Material. The determination of 2-FAL in solution was carried out at the maximum adsorption wavelength of 2-FAL, 275 nm. It was determined by a V-750 double beam spectrophotometer (Jasco, Easton, MD, USA) equipped with 1 cm quartz cells. A typical standardization curve in water is (concentration in mol L^−1^ (M): A_275_ = 1.80(1) × 10^4^ c + 0.003(9) (LOD = 1.7 × 10^−7^ M).

The UV spectra of other furanic compounds, possibly interfering substances, show adsorption maxima at values near to that of 2-FAL (see Supplementary Material). The standardization curves at the respective maximum absorbance of two substances are:

5-Hydroxymethyl-2-furaldehyde (HMF) at λ = 286 nm: A_286_ = 1.890(2) × 10^4^ c + 0.001(1) (LOD = 1.6 × 10^−7^ M).
Furfuryl alcohol (FA) at λ = 217 nm: A_217_ = 0.992(2) × 10^4^ c + 0.003(9) (LOD = 3.0 × 10^−7^ M).

The limit of detection (LOD) is calculated as three times the standard deviation of the ordinate at the origin, divided by the sensitivity. The LOD is higher when the sample solution is the supernatant of the binding and extraction experiments, since some adsorbing substance is co-extracted from MIPs.

#### 2.6.2. Batch Equilibration

Batch equilibration experiments were performed by contacting weighed amounts of polymer (usually 0.04 g) with measured volumes (usually 3 mL) of solution with known concentrations of 2-FAL. The ratio of the solution volume to mass of adsorbing MIP (g) is 75 mL_sol_/g_MIP_. The equilibration time must be sufficiently long to achieve the binding equilibrium, typically 14 h. The analysis of the supernatant solution at equilibrium condition has been performed by UV spectrophotometry, after separation of the liquid phase by centrifugation (usually 10 min at 4000 rpm). The separated MIP is contacted with ethanol 96% for re-extraction of the adsorbed 2-FAL. One extraction with 3 mL ethanol is usually sufficient to extract the adsorbed 2-FAL.

#### 2.6.3. Voltammetric Determination

Voltammetric determinations with the MIP-modified SPCs were made with a portable potentiostat (Palm-Sens, Utrecht, The Netherlands) equipped with the PalmSens PC ver.2.33 software. Square wave voltammograms (SWV) were usually obtained at the following conditions: Estart = 0.000 V; Eend = −1.9 V; Estep = 0.03 V; Epulse = 0.066 V; f = 31.0. All the electrochemical experiments were made in an air conditioned room at 25 °C, in non-deaerated solutions NaCl 0.1 M (10 mL of solution, for a ratio of the solution volume to mass of adsorbing MIP of 1250 mL_sol_/g_MIP_).

#### 2.6.4. SPR Determination

The SPR determinations were made by the D-shaped POF-MIP-SPR sensor. The experimental setup consists of a halogen lamp (HL–2000–LL, Ocean Optics, Dunedin, FL, USA) illuminating the SPR-POF-MIP sensor and a spectrum analyzer (USB2000+UV–VIS spectrometer, Ocean Optics) connected to a computer. The spectral emission of the lamp ranges from 360 nm to 1700 nm and the measurements were performed from 300 nm to 1050 nm. Measurements were performed by dropping a small volume, usually 40 μL, of the appropriate solution on the platform and incubating for 5 min. The light transmission spectrum was obtained. The spectra were normalized to the transmission spectrum obtained at the same platform, before the MIP deposition (bare platform), in the air, since in this dielectric not any plasmon resonance is expected to take place due to the low RI of air [11]. The transmission minimum corresponds to the resonance wavelength The signal is given by the variation of the resonance wavelength (Δλ, nm) with respect to the resonance wavelength of the blank solution, due to the refractive index variation of the polymeric layer upon the combination of the substrate.

### 2.7. Evaluation of the Binding Parameters of 2-FAL on MIP

#### 2.7.1. Binding Parameters by Batch Equilibration

The binding parameters are usually evaluated from the adsorption isotherms by applying some adsorption models in order to fit the experimental data. One of the most commonly applied model is the Langmuir isotherm [29,30] which relies on the assumption that binding takes place by combination of the substrate A with receptor sites in the solid (R), according to the following equilibrium:

(1) $$A+R↔AR\;K_{A}=\frac{\left[RA\right]}{\left[R\right]\left[A\right]}$$

R represents the receptor sites for A, obtained by the molecular imprinting method with a given affinity and at concentration c_R_. [R] is the concentration of the free sites and [RA] is the concentration of the sites occupied by the target substance A. These concentrations are referred to the polymer phase (in mmol/g_MIP_).

The Langmuir sorption isotherm is:

(2) $$\left[RA\right]=\frac{c_{R}K_{A}\left[A\right]}{1+K_{A}\left[A\right]}$$

Sometimes the Langmuir-Freundlich equation is used to account for the possible heterogeneity of the binding sites, and in this case an exponential m, the heterogeneity index, is introduced to account for the heterogeneity [29,30]. The parameters of Equation (2), included the heterogeneity index, can be determined by non-linear regression, available in the common statistical packages. In the present study the Hill equation from the software OriginPro 2016 b9.3.226 has been used.

Alternatively, the relation can be expressed in the Scatchard linear format, which has been widely employed for the evaluation of the parameters:

(3) $$\frac{\left[RA\right]}{\left[A\right]}=-K_{A}\left[A\right]+K_{A}c_{R}$$

This model well fit the saturation phenomena which are always observed when a sufficiently high amount of A is adsorbed on the MIP i.e., when [A] >> 1/K_A_, and [A] = c_R_. Equation (2) and the derived Equation (3) hold when only one kind of interaction sites is present in MIP, however different kinds of sites with different affinities for A can be present in the non-covalent MIPs [20], due to their inherent heterogeneity. The presence of multiple classes of sites is indicated by a curvature of the Scatchard plot. In this case, when for example two kinds of sites are present, the bi-Langmuir model can be applied [29,30] considering at the same time the sites with higher association constant (K_A2_) and site concentration c_R2_, and those with lower association constant (K_A1_) and site concentration c_R1_. The corresponding relationship is

(4) $$\left[RA\right]=\frac{c_{R2}K_{A2}\left[A\right]}{1+K_{A2}\left[A\right]}+\frac{c_{R1}K_{A1}\left[A\right]}{1+K_{A1}\left[A\right]}$$

In this case the Scatchard plot (Equation (3)) should be composed of two straight lines at the extreme concentrations. Usually not more than two different kinds of sites are detectable, so a bi-Langmuir model is sufficient for the sorption characterization in a given concentration range. The adsorption on the corresponding NIP can be examined by the same model.

#### 2.7.2. Binding Parameters by Sensors

The signal obtained from a sensor is usually directly proportional to the analyte concentration in the layer near the transductor. In this work the signal given by the electrochemical sensor is the peak current (i_p_) obtained by square wave voltammetry (SWV), and that by the SPR sensor is the resonance wavelength variation Δλ. In both cases the relation between the concentration of the adsorbed target, [RA], and the signal S is:

S = k [RA](5)

The constant k is different for different transduction methods, i.e., k_EC_ in the case of the electrochemical transduction and k_SPR_ in the case of SPR transduction. According to Equation (5), the Langmuir equation (Equation (3)) can be transformed in the following way:

(6) $$S=\frac{k\;c_{R}K_{A}\left[A\right]}{1+K_{A}\left[A\right]}$$

Equation (6) is a binding isotherm, in which a signal related to the concentration in the MIP phase is measured instead of the adsorbed concentration. The maximum signal i_pmax_, in the case of electrochemical transduction, or Δλ_max_ in the case of SPR transduction, is obtained when [A] >> 1/K_A_, and S_max_ = kc_R_. Similarly to the isotherm obtained by batch equilibration method, a common linearized form of the Equation (6) is the Scatchard plot:

(7) $$\frac{S}{\left[A\right]}=-K_{A}\left[A\right]+K_{A}kc_{R}$$

From the binding isotherms obtained by sensors it is possible to evaluate K_A_ but not c_R_, at least if k (the proportionality constant of the signal) is unknown.

For two kinds of sites with association constants K_A1_ and K_A2_ the following relationship holds

(8) $$S=\frac{k\;c_{R2}K_{A2}\left[A\right]}{1+K_{A2}\left[A\right]}+\frac{k\;c_{R1}K_{A1}\left[A\right]}{1+K_{A1}\left[A\right]}$$

## 3. Results

In the present investigation a MIP obtained by a commonly used method is examined i.e., a MIP obtained for 2-FAL as the template by non-covalent imprinting. It is expected that sites with different affinities are present, which are investigated considering different concentration levels, using various detection methods. For concentrations at around 10^−4^–10^−3^ M batch equilibration experiments have been performed. In this way both the association constant and the adsorption capacity could be evaluated. The two approaches based on sensors with different transduction principles, i.e., the electrochemical and optoelectronic transduction, have been applied to study the adsorption at lower concentrations.

### 3.1. Binding of 2-FAL to MIP by Batch Equilibration

**Figure 1:**
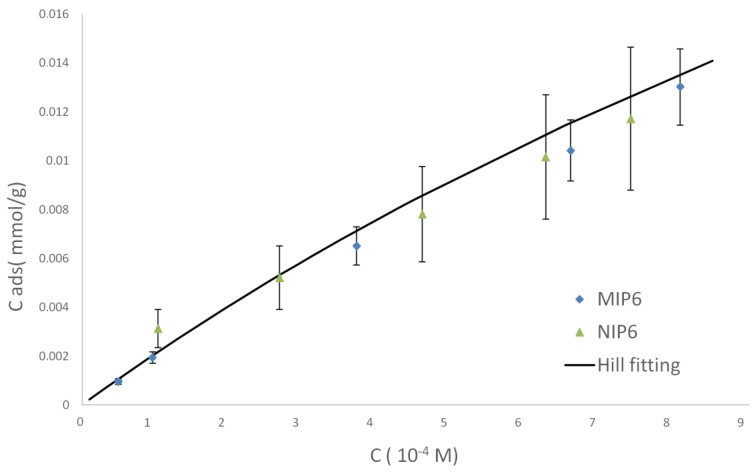
Binding isotherms of 2-FAL on MIP and NIP from water, at 75 mL_sol_/g_MIP_.

Batch equilibration is the most widely used method to characterize the sorption of a molecule on a solid. Here it has been applied to obtain the sorption isotherms in the concentration range from 5 × 10^−5^ M to 90 × 10^−5^ M. This is the range in which UV measurement at 275 nm can be used for 2-FAL concentration determination. Typical binding isotherms of 2-FAL on MIP-2FAL and on the corresponding NIP are reported in Figure 1.

It can be seen that a linear relationship between the bound ([RA]) and free ([A]) concentration is obtained approximately only up to 4 × 10^−4^ M, while the slope decreases at higher concentration indicating that the polymer is approaching saturation [29]. Practically the same curve is obtained for MIP and NIP, which is an evidence that at these concentration levels the adsorption is not driven by the interaction with the imprinted sites. By applying the Langmuir model (Equation (2)) in the Scatchard form (Equation (3)), a single straight line is obtained indicating that only one kind of binding sites can be detected in the considered concentration range. *K*_A_ has been found to be 3.9(7) ×10^2^ M^−1^ with a maximum adsorbable amount (c_R_) of 0.053(20) mmol/g_MIP_. Because the prevailing adsorption mechanism seems not to involve imprinted sites, it is expected that furanic compounds with molecular structure similar to that of 2-FAL are adsorbed as well. Actually, it has been found that furanic compounds as 5-hydroxymethyl-2-furaldehyde (HMF) and furfuryl alcohol (FA) are adsorbed on MIP-2FAL. At the considered concentration, i.e., around 8 × 10^−4^ M, the bound-to-free ratio (B/F, expressed as amount of polymer bound and solution free compound at equilibrium) is 0.21 for 2-FAL, 0.12 for HMF and 0.58 for FA. Notice that one non-template molecule as FA has a higher bound-to-free ratio than the template 2-FAL, which shows that interactions different from those with the imprinted sites prevail at the considered conditions [40]. It can be guessed that the carboxylic groups from MAA present at the polymer surface not structured in any imprinted site are responsible for the binding of the considered furanic compounds.

### 3.2. Investigation of the Binding of 2-FAL to MIP by the Electrochemical SPC-MIP Sensor

**Figure 2:**
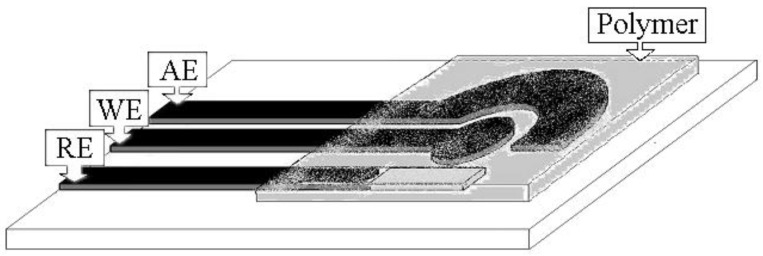
Schematic view of screen-printed cell modified by a polymeric layer, either MIP (SPC-MIP) or NIP (SPC-NIP). WE: working electrode (graphite ink); AE: auxiliary electrode (graphite ink); RE: reference electrode (silver ink).

In order to characterize other binding sites possibly present in the considered MIP, an approach based on electrochemical sensing has been considered. The device used for this investigation is schematically reported in Figure 2. The MIP layer is obtained in a very simple way by dropping a small volume of prepolymeric mixture over the whole cell and in situ thermal polymerization.

The signal obtained by this technique, i.e., the peak current by SWV, depends on the concentration of the template in the polymeric receptor layer, which in turn depends on the activity in the solution phase. The binding isotherms were treated according to the Scatchard equation (Equation (7)), in order to evaluate the association constant, *K*_A_, and the maximum signal, i_pmax_ = kc_R_.

**Figure 3:**
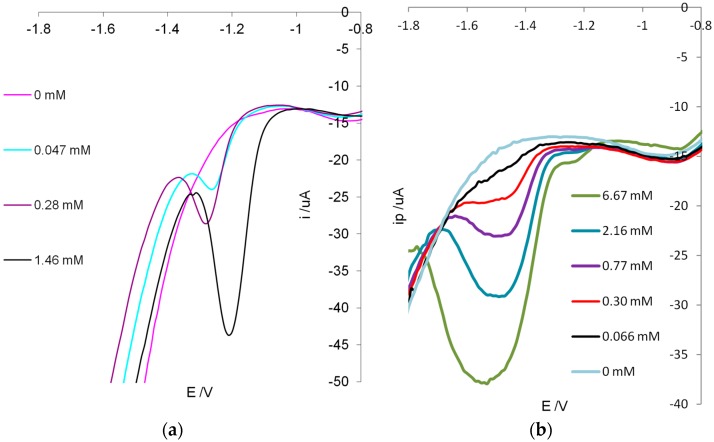
Voltammograms in NaCl 0.1M water solution. (**a**) bare platform, (**b**) SPC-MIP5 SWV conditions: E_s_ = 0.01 V, E_p_ = 0.025, f = 25 p/s.

Sensing is based on the electrochemical activity of 2-FAL, which is reduced to furfuryl alcohol at potentials around 1–1.5 V. While this reaction is well known at the mercury electrode, it has been claimed that it does not takes place at other electrodes such as the glassy carbon (GC) and bare carbon paste electrode (CPE) [39]. In the present work, screen printed cells (SPCs) with graphite ink working electrode have been considered as the electrochemical platform for their reproducibility and low cost. It is relevant that the sensor preparation is fast and easy, since SPCs have a flat surface, in which a reproducible MIP layer can be easily deposited by drop coating, and subsequently polymerized by thermal initiation in an oven, as previously described for other analytes [36,37,38]. The deposition is particularly easy since it has been shown that the whole cell can be covered with MIP, with good results in terms of reproducibility and selectivity, by a simple drop coating procedure. The experimental determinations have been performed by square wave voltammetry (SWV) evaluating the peak current in function of the template concentration in the solution phase. For comparison typical voltammograms obtained at the bare screen printed cell and at MIP modified cell at different concentrations of 2-FAL are reported in Figure 3.

The investigation of the electrochemical reduction mechanism is outside the aim of the present investigation, but it is important that an electrochemical signal depending on the concentration of 2-FAL is obtained at the screen printed cell, as seen in Figure 3.

In the case of MIP-modified sensor the reduction of 2-FAL takes place at lower potential than at the bare cell, and the reduction peaks are larger. This could indicate that 2-FAL in the polymer phase is linked to sites with somewhat different affinity. Despite of the peak broadening, the peak current (i_p_) is related to the concentration of 2-FAL in solution, and can be used to plot the binding isotherms.

**Figure 4:**
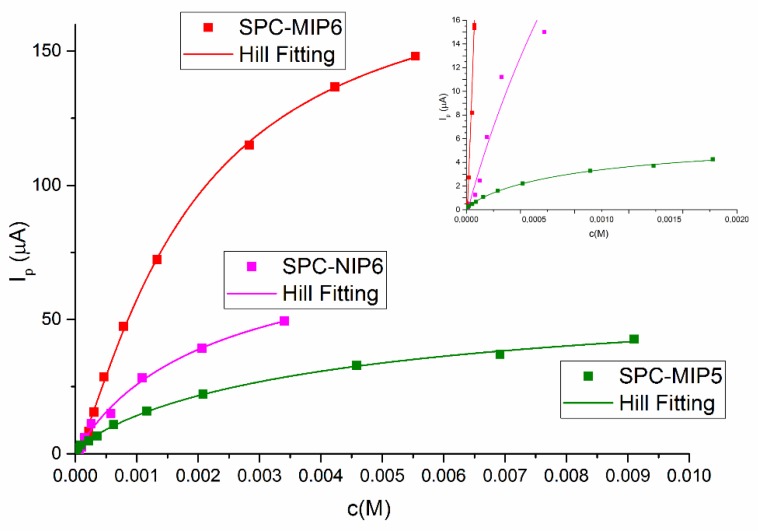
Binding isotherms of 2-FAL reporting the voltammetric response by SWV at the following conditions: E step = 0.03 V; E pulse = 0.065 V; f = 28 pulse/s. Sensor: SPC-MIP6 (red line), SPC-MIP5 (green line), SPC-NIP6 (magenta line).

Some binding curves of MIP are reported in Figure 4, together with that obtained with the corresponding non-imprinted polymer (NIP6).

**Figure 5:**
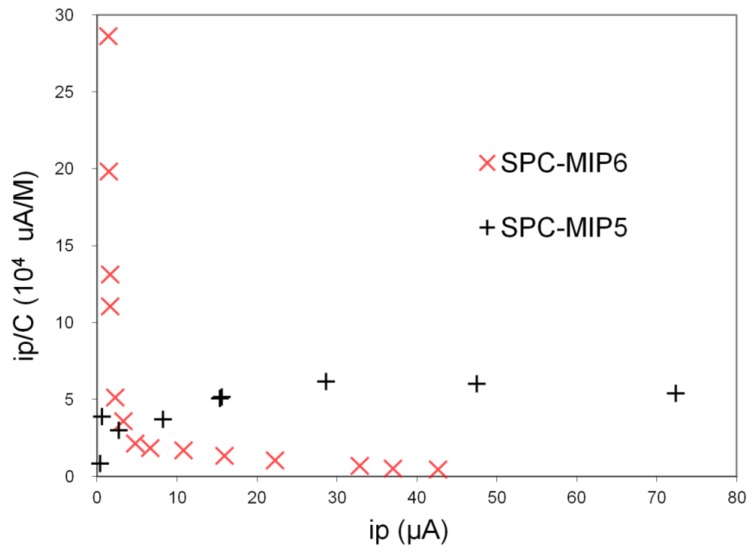
Scatchard plots for binding of 2-FAL by SPC-MIP6 and SPC-MIP5 sensors based on SWV at the following conditions: E step = 0.03 V; E pulse = 0.065 V; f = 28 pulse/s. NaCl 0.1 M water solution.

The binding isotherms are not straight lines but rather they present a saturation behavior at higher concentration. This suggests that binding of 2-FAL to the corresponding MIP takes place according to the Langmuir or bi-Langmuir model. The corresponding Scatchard plots (Equation (7)) for SPC-MIP6 and SPC-MIP5 are reported in Figure 5. It is seen that in the case of SPC-MIP6 only the points at high concentration, i.e., higher than 4 × 10^−4^ M, giving signals higher than about 30 μA, are fit by the Scatchard model. At the higher concentrations K_A_ was evaluated as 4.1(3) × 10^2^ M^−1^, which is similar to that found by the batch equilibration procedure in the same concentration range. From the Scatchard model the maximum signal obtainable at saturation (kc_R_, see Equation (7)) is evaluated to be 213(22) μA. From Figure 4 it is seen that the corresponding NIP (SPC-NIP6) too is able to adsorb 2-FAL, but at a lower extent. Here too the concentrations which can be fit by the Scatchard model are those higher than about 2 × 10^−4^ M, for which the association constant is 5.4(5) × 10^2^ M^−1^ not significantly different from that obtained by the sensor SPC-MIP6, indicating that the adsorption mechanism predominating at higher concentration does not involve the imprinted sites. For NIP, the maximum signal at saturation evaluated by the Scatchard plot was 75(36) μA, about three times lower than that obtained by the MIP based sensor.

By batch equilibration the binding of 2-FAL was the same for MIP and NIP at least up to 9 × 10^−4^ M. This can be ascribed to the much higher contact surface of solution and MIP in the batch equilibration experiments. In the case of the electrochemical sensor, some differences in the porosity could produce the saturation of NIP at lower adsorbed amounts of 2-FAL than MIP. A different sensor obtained by a lower amount of prepolymeric mixture, was considered too, SPC-MIP5. It was obtained by in situ polymerization of only 3 μL of prepolymeric mixture. For comparison, the binding isotherm of SPC-MIP5 is reported in Figure 4 and the Scatchard plot in Figure 5.

In Figure 5 it is seen that the SPC-MIP5 sensor, with a thinner polymeric layer, shows a typical bi-Langmuir behavior, with two kinds of sites involved in the binding of 2-FAL. One of them predominates at concentration higher than about 4 × 10^−4^ M (sites 1), and the other at concentrations lower than 2 × 10^−5^ M (sites 2). The parameters characterizing the binding of 2-FAL to the two kinds of receptors detected, obtained by the bi-Langmuir model considering the limiting slopes, are reported in Table 1:

The sites with lower affinity (*K*_A1_ = 3.1 × 10^2^ M^−1^) are similar to those evaluated with the SPC-MIP6 sensor as far as the association constant is concerned, but the signal corresponding to saturation (51 μA) is about 4.5 times lower than that obtained with the SPC-MIP6 sensor examined above (244 μA), which was built up with an higher amount of MIP, about three times. This is roughly proportional to the amount of MIP used for the formation of the receptor layer. The association constant is similar to that obtained by the batch equilibration procedure at the same concentration level, i.e., higher than 10^−4^ M. Actually the concentration range for which a useful signal is obtained is the same.

Sensor SPC-MIP5 makes it possible to detect sites at higher affinity (3.6 × 10^5^ M^−1^) too, which are involved in 2-FAL binding at concentration lower than about 2 × 10^−5^ M. The higher association constant is two magnitude orders higher than that evaluated at lower concentration. The combination with these sites generates a maximum signal (kc_R_) as low as 2.4 μA, about 20 times lower than the maximum signal obtainable by combination with the weaker sites, which could be due to the lower concentration of the high affinity sites [29].

### 3.3. Investigation of the Binding of 2-FAL to MIP by an SPR Sensor

**Figure 6:**
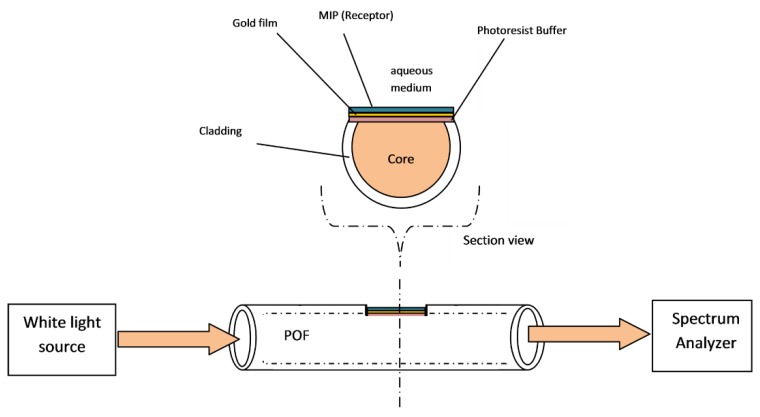
SPR sensor based on D-shaped POF (D-shaped POF-MIP-SPR).

The receptor layer binding properties are often investigated by SPR measurement based on prism (Kretschmann configuration). Compared to this “classical” configuration, the D-shaped POF based SPR platform has the advantage of not requiring an expensive dedicated apparatus. A schematic view of the sensor is reported in Figure 6.

The possibility of performing measurements in a particularly convenient way for practical application, i.e., in a few μL drop, is offered by the shape of the optical platform here proposed, which presents a flat surface and can be easily maintained in a horizontal position.

**Figure 7:**
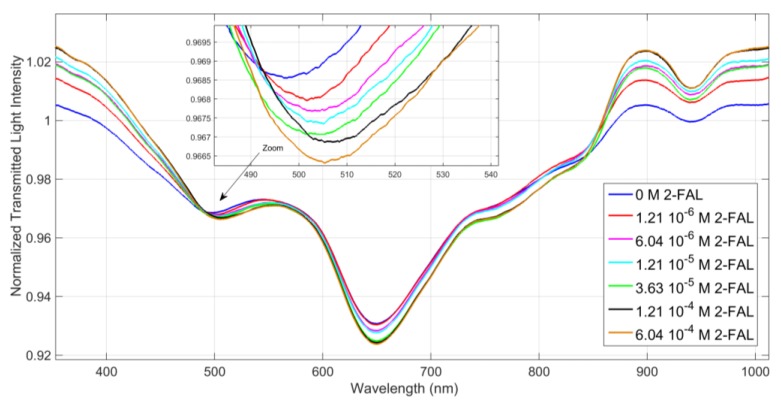
Normalized spectra (against the bare optical platform in air) of the D-shaped POF-MIP-SPR sensor in 75%water-25%ethanol, at different concentrations of 2-FAL in the range 1 × 10^−6^ M to 6 × 10^−4^ M.

As an example some spectra obtained with the D-shaped POF-MIP-SPR sensor are reported in Figure 7. They were acquired in 75%water-25%ethanol solution at different concentration of 2-FAL, by contacting a drop of sample with the MIP receptor layer for 5 min before the spectrum registration. One resonance dip is present at around 500 nm, depending on the concentration of 2-FAL. The resonance wavelength shift with increasing concentration of 2-FAL is clearly seen in the inset.

**Figure 8:**
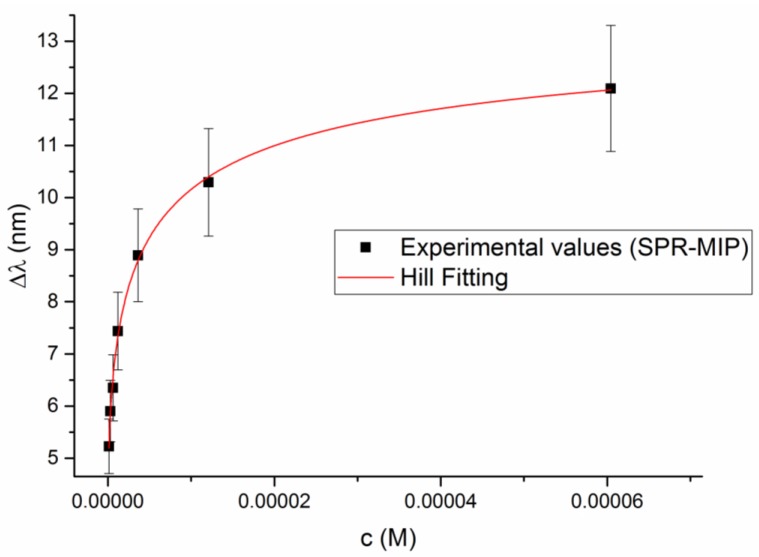
Sorption isotherm of 2-FAL on MIP, obtained from the resonance wavelength shift observed in Figure 7.

The response is obtained for 2-FAL concentrations much lower than those for which an electrochemical response was acquired. The sorption isotherm at about 500 nm for 2-FAL is shown in Figure 8. The continuous curve was calculated with the parameters obtained by the Hill fitting [12]: K = 1.1(3) × 10^7^ (M^−1^); sensitivity at low concentration = 5(2) × 10^7^ (nm × M^−1^), n = 1.

**Figure 9:**
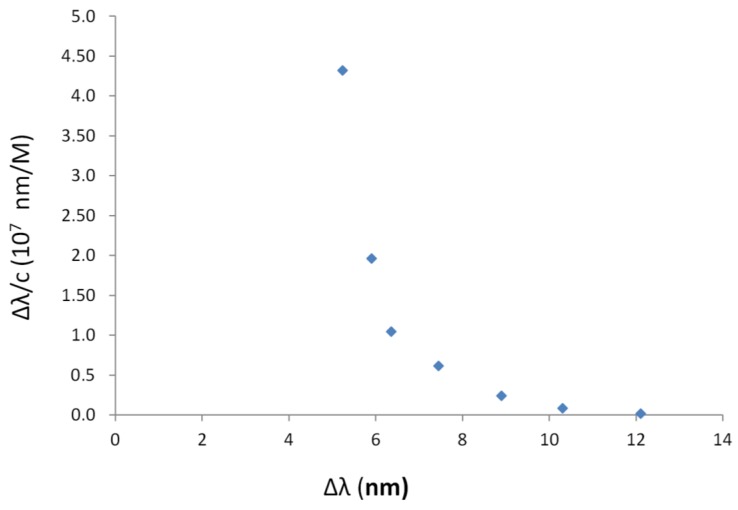
Scatchard plot (Equation (7)) for the data from the spectrum in Figure 7. Adsorption of 2-FAL on MIP-DVB from water, monitored by the D-shaped POF-MIP-SPR sensor.

A good fit of the data is obtained by the Hill model, which can be of great help for quantification purposes. However from the Scatchard plot (Equation (7)) it is clearly seen that two different complexes are formed in the concentration range 1.0 × 10^−7^ M–1.2 × 10^−5^ M as shown in Figure 9. The binding parameters of the two sites involved in the combination with 2-FAL in the considered concentration range are reported in Table 2.

The association constant (*K*_A2_) of 2-FAL for the sites at low affinity is not significantly different from that obtained by the electrochemical platform at a similar concentration range. For this reason these sites are named “sites 2” as sites 2 detected by the electrochemical sensor. In addition sites with much higher affinity are detected too by the SPR sensor (sites 3). The maximum signal is very similar for the two kinds of interaction sites, despite of the fact that the sensitivity is much lower. The same kind of sensor prepared using NIP instead of MIP did not give any resonance wavelength shift at the same 2-FAL concentration range. This indicates that the sites at high affinity have also a high selectivity.

Different substances with molecular structure very similar to that of 2-FAL, i.e., furanic compounds as 5-hydroxymethyl-2-furaldehyde (HMF) and furfuryl alcohol (FA) were considered for testing the selectivity, as reported in the case of batch equilibration. There was not any adsorption of the considered substances on the MIP, as demonstrated by the fact that not any resonance wavelengths shift was observed. This shows that the sites with higher affinity are at the same time those with the higher selectivity.

## 4. Discussion

**Figure 10:**
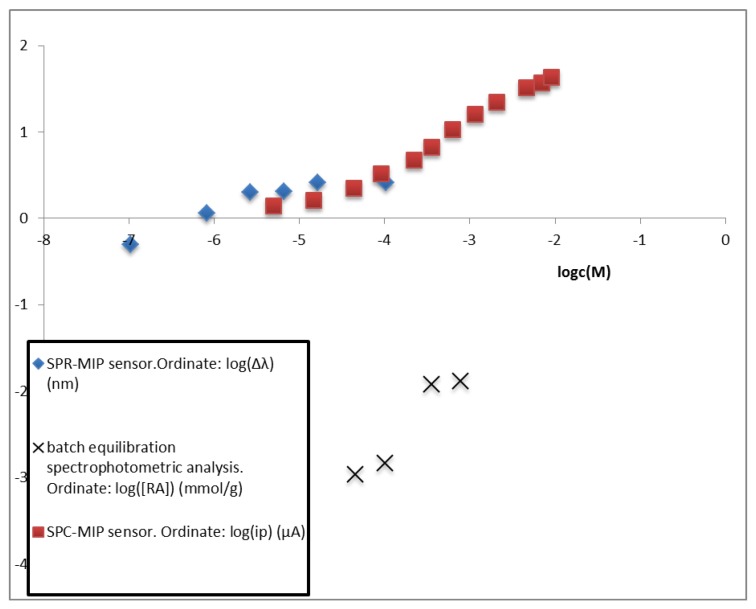
Adsorption isotherms obtained by different methods: optical SPR-MIP sensor, electrochemical SPC-MIP sensor and by batch equilibration and spectrophotometric analysis.

Adsorption isotherms in different concentration ranges have been obtained by the different characterization techniques proposed in the present investigation. For comparison, the binding isotherms of the same MIP with the different methods are reported in Figure 10 using the log-log relationship [41,42] in order to clearly compare the whole considered concentration range from 10^−7^ to 10^−2^ M. The depending variables are different, according to the different method used and as specified in the legend.

Only in the case of the batch equilibration method a straight line with slope only slightly lower than 1 is obtained, indicating that conditions far from the saturation [19,20,29] are considered in the reported experiments. Notice that in this case the ratio of the solution volume to the mass of adsorbing MIP is 75 mL_ext_/g_MIP_. Moreover, the Scatchard plot (Equation (3)) shows the presence of only one kind of binding sites, whose association constant and concentration (c_R_) have been evaluated, as reported above. These results do not imply that other classes at higher affinity are not present in the considered MIP, but only that, if present, they are at lower amount than the non-specific sites.

On the contrary, a saturation is clearly observed in the binding isotherm by electrochemical sensor at concentrations higher than about 3 × 10^−4^ M (see Figure 4). The difference can be ascribed to the fact that in this case the ratio of the solution volume to the mass of adsorbing MIP is about 1250 mL_sol_/g_MIP_, i.e., much higher than that in the batch experiment. At lower concentration a behavior approaching saturation is observed too, since the log-log isotherm in the lower concentration range has a slope well below 1. This indicates the existence of at least two kinds of binding sites, so that the bi-Langmuir model has been applied to evaluate the respective affinities as reported in Table 1. It is relevant that the same affinity, indicated as K_A_ in batch equilibration and K_A1_ in electrochemical sensing, is evaluated in the same concentration range, using completely different investigation methods and conditions.

Due to the better sensitivity and reproducibility of the electrochemical sensing compared to the batch equilibration, other imprinted sites with higher affinity for 2-FAL are detected too, and characterized by their association constant, K_A2_. It is worth noticing that this part of the log-log binding isotherm has a very low slope, which indicates that the detected sites are near to the saturation (see the kc_R2_ value in Table 1). The D-shaped POF-MIP-SPR sensor responds to even lower concentrations than the electrochemical one, partially overlapping the concentration range detected by the electrochemical sensor. As in that case, both the log-log isotherm (Figure 10) and the Scatchard plot (Figure 9) reveals the presence of two different kinds of sites for 2-FAL, one of which, that detected in the same concentration range, is very similar to that determined by the electrochemical sensor. In addition, the SPR sensor allowed to investigate a lower concentration range, corresponding to sites with even higher association constant, K_A3_ (see Table 2).

## 5. Conclusions

In this research different methods for the investigation of the binding properties of a synthetic receptor, a molecularly imprinted polymer (MIP-2FAL), have been applied with the aim of determining its binding properties in wide concentration ranges. It has been found that this investigation can be conveniently performed using different sensing devices with different detection ranges. With respect to the batch equilibration methods, based on the determination of the target substance left in solution after equilibration, the methods based on sensing devices are convenient not only in terms of amount of binding material required for the investigation, for example a few mg of MIP instead of tens of mg, and of the time required for obtaining the information, but also because they make it possible to investigate the interactions at lower concentrations due to their high sensitivity. This strongly depends on the transduction method considered. It has been shown that different concentration ranges can be detected by different transduction methods and that when the concentration ranges overlap, sites with similar affinity are detected. This is a strong indication that actually sites with different affinity are present in the considered MIP. The use of SPR transduction allowed lower concentrations to be investigated than those by voltammetric sensor, even if in the present investigation the measurements were performed with a very simple apparatus based on POF instead of Kretschman configuration on prism. It has been found that at least three kinds of sites, with association constant ranging from about 5 × 10^2^ M^−1^ to 3 × 10^7^ M^−1^, are present in the MIP-2FAL here examined as a proof of principle. The sites distribution can be different in other MIPs, however the existence of heterogeneous sites in the same MIP, particularly those obtained by non-covalent imprinting, agrees well with a plethora of previous findings [19,20,21,29]. The reasons for the heterogeneity have been widely discussed in a number of review papers, for example by Garcia-Calzon and Diaz-Garcia [30]. No information about the structure of the heterogeneous sites can be obtained from the association constants determined by the approaches of the present investigation. It can be guessed for example that sites can be formed by a different number of functional monomers or with different rigidity of the polymer around the site [20,30]. In the case here considered the functional monomer was present in large excess with respect to the template. Thus it can be expected that a large part of the functional monomer is free, and contribute to the nonspecific binding, as it has been actually found. Both electrochemical and SPR platforms here proposed are easy and fast to prepare, so they can be considered as useful tools for characterization of the binding properties of the receptor in an easy and rapid way, each of them in a particular concentration range.

## Figures and Tables

**Table 1:** Binding parameters of 2-FAL on MIP5 determined by the sensor SPC-MIP5 by Scatchard plot, and sensing parameters.

	*K*_A_ (M^−1^)	kc_R_ (μA)	Sensitivity (μA/M) at Low Concentration	LOD (M) ^1^
Site with high affinity (sites 2)	2(1) × 10^5^	2(2)	6(2) × 10^5^	7.6 × 10^−7^
Sites with low affinity (sites 1)	3.1(9) × 10^2^	51(1)	1.60(2) ×10^4^	

^1^ LOD obtained as 3xs of the ordinate at the origin of the dose response curve by SPC-MIP5 divided by the sensitivity at low concentration.

**Table 2:** Binding parameters of 2-FAL on MIP determined by the D-shaped POF-MIP-SPR sensor SPR-MIP by Scatchard plot, and sensing parameters.

	*K*_A_ (M^−1^)	kc_R_ (nm)	Sensitivity at Low Concentration (nm/M)	LOD (M)^1^
Sites at high affinity (sites 3) (c up to 6 10^−7^ M)	3.0(4) 10^7^	7(2)	2.0(2) 10^8^	5 × 10^−9^
Sites at low affinity (sites 2) (c =3-60 10^−6^ M)	1.7(2) 10^5^	6(1)	9.7(6) 10^5^	

^1^ LOD obtained by dividing the resolution of the spectrometer (1.5 nm) by the sensitivity at low concentration.

## Supplementary Materials

The following are available online at https://www.mdpi.com/1424-8220/19/6/1344/s1, Figure S1. UV spectra of 2-FAL, in 0.75H_2_O:0.25EtOH. 3.02 × 10^−5^ M (light green); 6.04 × 10^−5^ M (brown); 9.06 × 10^−5^ M (blue); 12.08 × 10^−5^ M (green), Figure S2. UV spectra of HMF, in 0.75H_2_O:0.25EtOH. 1.95 × 10^−^^5^ M (light green); 3.91 × 10^−^^5^ M (brown); 5.96 × 10^−^^5^ M (blue); 7.81 × 10^−^^5^ M (green), Figure S3. UV spectra of FA, in 0.75 H_2_O:0.25 EtOH. 2.89 × 10^−^^5^ M (light green); 5.79 × 10^−^^5^ M (brown); 8.69 × 10^−^^5^ M (blue); 11.58 × 10^−^^5^ M (green).
