# Supplementary material for: Sensing by Molecularly Imprinted Polymer: Evaluation of the Binding Properties with Different Techniques
Source: Sensors (Basel). 2019 Mar 18;19(6):1344. doi: 10.3390/s19061344 (PMC6470915; doi:10.3390/s19061344)
Supplement: Supplementary file 1 [file sensors-19-01344-s001.pdf]

Supplementary Material

Sensing by Molecularly Imprinted Polymer: Evaluation of the Binding Properties with Different Techniques

Maria Pesavento [1,*], Simone Marchetti [1], Letizia De Maria [2], Luigi Zeni [3] and Nunzio Cennamo [3]

[1] Department of Chemistry, University of Pavia, 27100 Pavia PV, Italy; simone.marchetti01@universitadipavia.it
[2] Research on the Energetic System, 20134 Milan, Italy; Letizia.DeMaria@rse-web.it
[3] Department of Engineering, University of Campania Luigi Vanvitelli, 81031 Aversa, Italy; luigi.zeni@unicampania.it (L.Z.); nunzio.cennamo@unicampania.it (N.C.)
* Correspondence: maria.pesavento@unipv.it

UV spectra of furanic compounds

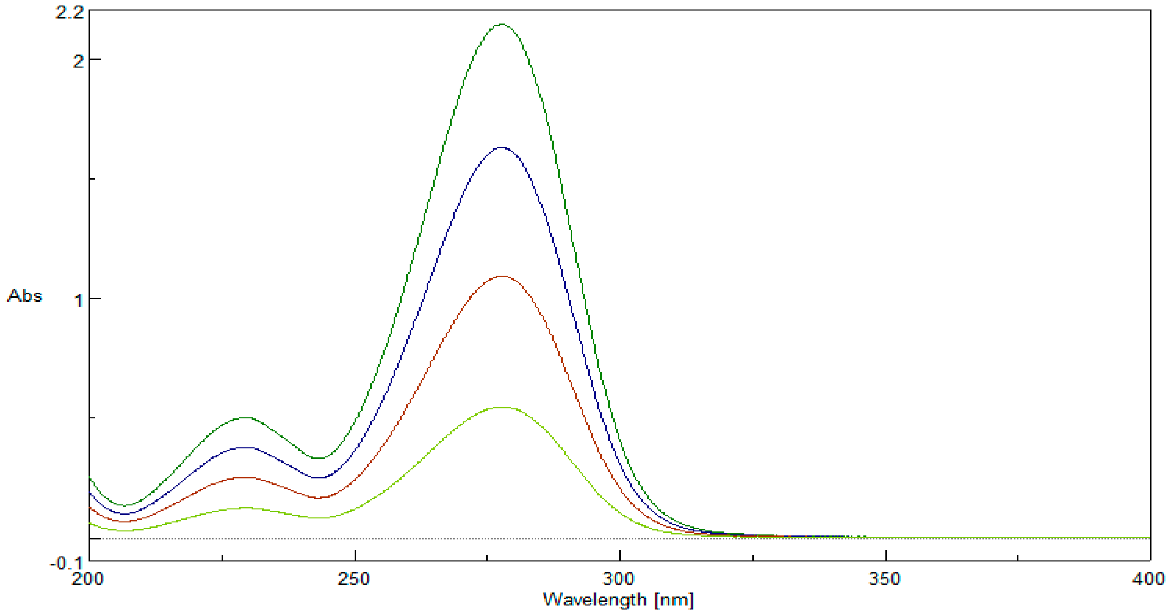

Figure S1. UV spectra of 2-FAL, in $0.75H_2O:0.25EtOH$. 3.02×10^{-5} M (light green); 6.04×10^{-5} M (brown); 9.06×10^{-5} M (blue); 12.08×10^{-5} M (green).

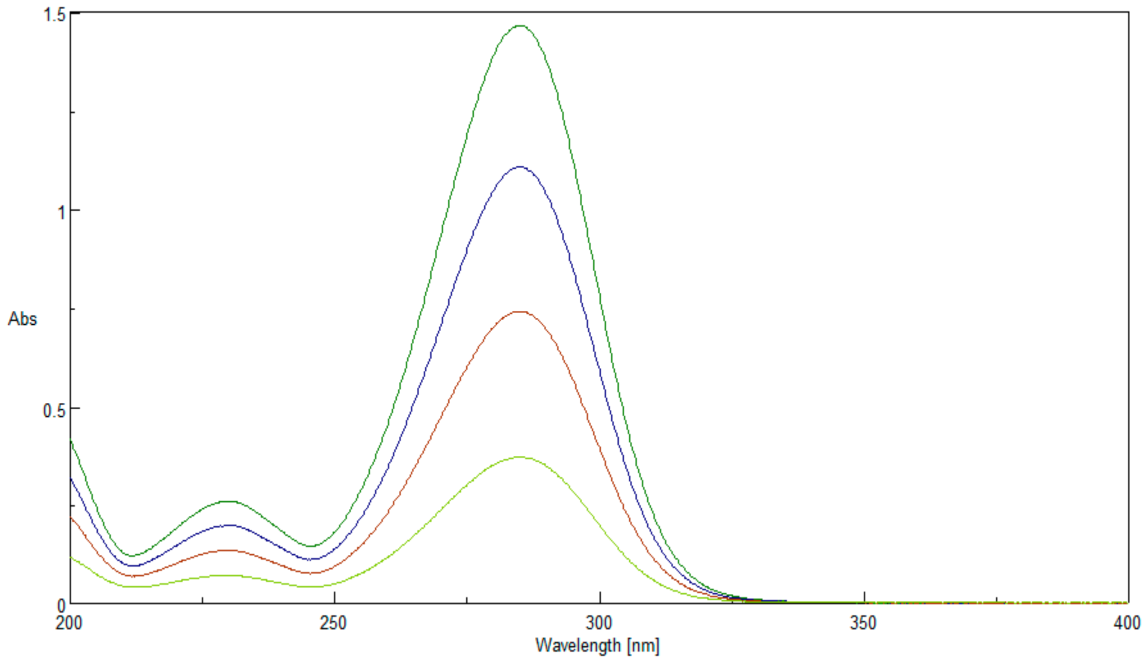

Figure S2. UV spectra of HMF, in 0.75H_2O:0.25EtOH. 1.95 × 10^{-5} M (light green); 3.91 × 10^{-5} M (brown); 5.96 × 10^{-5} M (blue); 7.81 × 10^{-5} M (green).

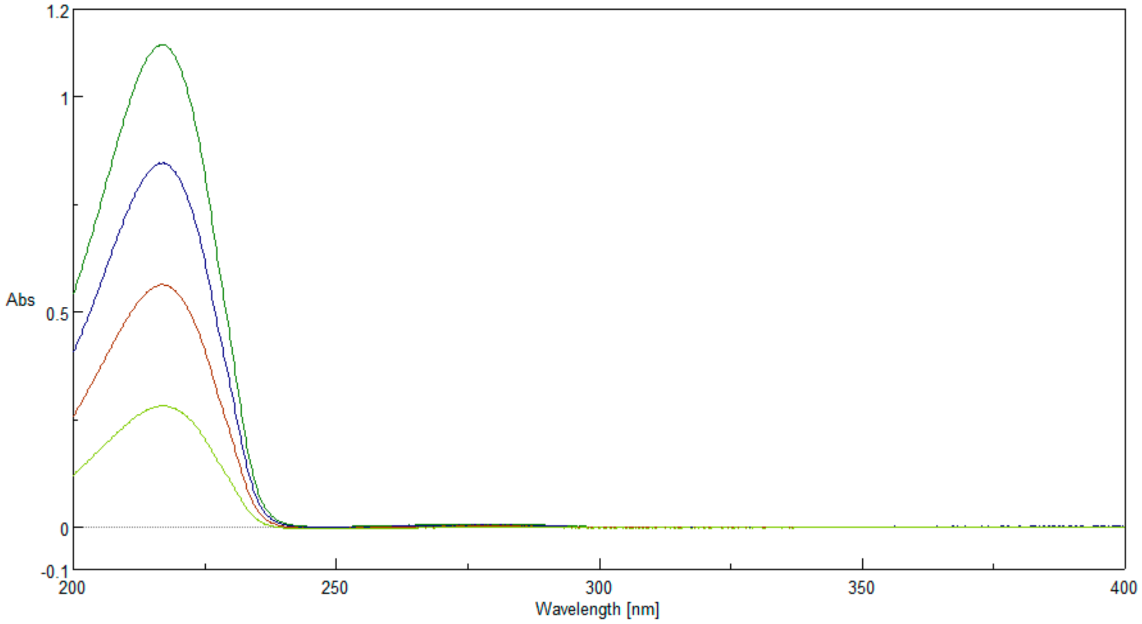

Figure S3. UV spectra of FA, in 0.75 H_2O:0.25 EtOH. 2.89 × 10^{-5} M (light green); 5.79 × 10^{-5} M (brown); 8.69 × 10^{-5} M (blue); 11.58 × 10^{-5} M (green).
